# Mitigating BeiDou Satellite-Induced Code Bias: Taking into Account the Stochastic Model of Corrections

**DOI:** 10.3390/s16060909

**Published:** 2016-06-18

**Authors:** Fei Guo, Xin Li, Wanke Liu

**Affiliations:** 1 School of Geodesy and Geomatics, Wuhan University, Wuhan 430079, China; fguo@sgg.whu.edu.cn (F.G.); lixinsgg@whu.edu.cn (X.L.); 2 Key Laboratory of Geospace Environment and Geodesy, Ministry of Education, Wuhan 430079, China; 3 Collaborative Innovation Center for Geospatial Technology, Wuhan 430079, China

**Keywords:** BeiDou, code bias variation, code-phase divergence, stochastic model, precise point positioning

## Abstract

The BeiDou satellite-induced code biases have been confirmed to be orbit type-, frequency-, and elevation-dependent. Such code-phase divergences (code bias variations) severely affect absolute precise applications which use code measurements. To reduce their adverse effects, an improved correction model is proposed in this paper. Different from the model proposed by Wanninger and Beer (2015), more datasets (a time span of almost two years) were used to produce the correction values. More importantly, the stochastic information, *i.e.*, the precision indexes, were given together with correction values in the improved model. However, only correction values were given while the precision indexes were completely missing in the traditional model. With the improved correction model, users may have a better understanding of their corrections, especially the uncertainty of corrections. Thus, it is helpful for refining the stochastic model of code observations. Validation tests in precise point positioning (PPP) reveal that a proper stochastic model is critical. The actual precision of the corrected code observations can be reflected in a more objective manner if the stochastic model of the corrections is taken into account. As a consequence, PPP solutions with the improved model outperforms the traditional one in terms of positioning accuracy, as well as convergence speed. In addition, the Melbourne-Wübbena (MW) combination which serves for ambiguity fixing were verified as well. The uncorrected MW values show strong systematic variations with an amplitude of half a wide-lane cycle, which prevents precise ambiguity determination and successful ambiguity resolution. After application of the code bias correction models, the systematic variations can be greatly removed, and the resulting wide lane ambiguities are more likely to be fixed. Moreover, the code residuals show more reasonable distributions after code bias corrections with either the traditional or the improved model.

## 1. Introduction

The Chinese BeiDou navigation satellite system (abbreviated as BDS, or BeiDou, or COMPASS) has already launched a regional navigation service by the end of 2012, and continues to develop a global system in the near future. As of October 2015, a total of 20 BeiDou satellites including six geostationary orbit (GEO), seven inclined geosynchronous orbit (IGSO), and seven medium-altitude Earth orbit (MEO) satellites have been launched, transmitting triple-frequency signals centered at B1 (1561.098 MHz), B2 (1207.14 MHz), and B3 (1268.52 MHz). However, it is worth mentioning that only 13 operational BeiDou satellites are presently available (5GEO + 5IGSO + 3MEO) for public use.

With the advent of BeiDou, a large amount of research has been dedicated to the aspects of precise orbit and clock determination [1,2,3], regional ionospheric and tropospheric delay modeling [4,5,6], differential code bias and timing group delay corrections [7,8,9,10], multipath effects analysis [11,12], triple-frequency ambiguity resolution [13,14], and precise positioning [1,15,16,17,18,19], *etc.* When it comes to the characteristics of BeiDou signals, Nadarajah *et al.* first discovered the BeiDou Inter-Satellite-Type Biases (ISTBs), and deeply investigated the impacts of such biases on real-time kinematic (RTK) positioning and mixed receiver attitude determination [20,21]. A recent study revealed that the code-phase divergences, which are absent for GPS, GLONASS, and Galileo, however, are commonly found in BeiDou IGSO and MEO satellites [22]. The systematic variation was first mentioned by Hauschild *et al.* [11], but the origin of the systematic variation was not identified. The same code bias variation was also detected by Montenbruck *et al.* through the analysis of multipath combination [23,24]. They found that the systematic bias is elevation-dependent, which varies by 0.4–0.6 m from horizon to zenith, particularly pronounced for the B1 signal. Since such systematic bias was consistently observed with other receivers and antennas it was, therefore, attributed to the transmitting satellites. More characteristics of the systematic bias variations were well discussed in Wanninger and Beer [22]. They identified significant differences between two groups of satellites (*i.e.*, IGSO and MEO) and among carrier frequencies (B1, B2, and B3). A correction model was then proposed to accommodate the satellite induced code bias variations, which is definitely an important contribution to the current BeiDou constellation. Nevertheless, the data used for modeling these variations is very limited. Observations from two 10-day intervals in March 2014 (DoY 070–079) and in June 2014 (DoY 160–169) were exclusively used. The long-term stability of the systematic biases could not be identified conclusively from such limited dataset. Consequently, the effectiveness of the mitigating model with fixed correction values may be a debatable issue. In addition, only the correction values on the specific nodes are provided, whereas the stochastic information, *i.e.*, the precision indexes, are completely missing in their correction model. Such precision indexes are, however, important for the stochastic model establishment in precise applications, such as precise point positioning (PPP). Ignoring the stochastic error of the corrections will lead to an overestimation of the actual precision of code observations. This is particularly pronounced in the case of inaccurate corrections are applied.

This paper deals with the same systematic bias variations as earlier described by Hauschild *et al.* [11], Montenbruck *et al.* [23,24], and Wanninger and Beer [22]. However, we use more datasets with a time span of almost two years (from January 2014 to October 2015) to model such orbit type-, frequency-, and elevation-dependent code biases. Moreover, the stochastic model of the corrections is considered as well. Following an analysis of the code bias variations and a brief introduction of the observation datasets used, we develop an improved correction model, which produce both correction values and their precision indexes. Afterwards, effectiveness validations for the proposed model are presented. Finally, we end with the conclusions.

## 2. Code Bias Variation Analysis

Providing dual- or triple-frequency GNSS observations, multipath (MP) effects are commonly assessed by a linear combination of code and carrier phase observables [12,25]. That is:

(1) $$MP_{i}(i,j)=P_{i}-\frac{f_{i}^{2}+f_{j}^{2}}{f_{i}^{2}-f_{j}^{2}}\lambda_{i}\varphi_{i}+\frac{2f_{j}^{2}}{f_{i}^{2}-f_{j}^{2}}\lambda_{j}\varphi_{j}$$

where $MP_{i}$ is the multipath combination on frequency i (i,j=1,2,3,i≠j), P and φ represent code range and carrier phase observables, f and λ are, respectively, the frequency and wavelength of the specific signal. If the phase noise and phase multipath are neglected, the MP combination mainly consists of code multipath, a constant ambiguity term which is a combination of the ambiguities of the two phase measurements, a combined signal delay, and thermal noise. Subtraction of the mean value from the measurements removes the phase ambiguities, which are constant if there are no cycle slips. The resulting time series of MP is dominated by code multipath and thermal noise.

The MP combination as shown in Equation (1) is an ionosphere-free and geometry-free combination. The linear coefficients are selected in such a way that ionospheric and tropospheric delay as well as all geometric contributions (e.g., clocks, orbits, and antenna movements, *etc.*) cancel out. Therefore, it can be used with both static and kinematic data. However, it is worth mentioning that the absolute values of multipath are unknown. Only variations of code multipath are available from the MP series.

For each code, there are at least two choices of MP combination using different frequencies among triple-frequency observations. Take BeiDou as an example; the code multipath on B1 (MP1) can be derived from Equation (1) using either (B1, B2) or (B1, B3) frequency combinations. However, one should be aware that the latter one is noisier than the former one due to a larger multiplication factor. Therefore, to keep a lower noise level, both MP1 and MP2 are derived from (B1, B2), and MP3 is computed from (B1, B3) combination in this paper.

**Figure 1:**
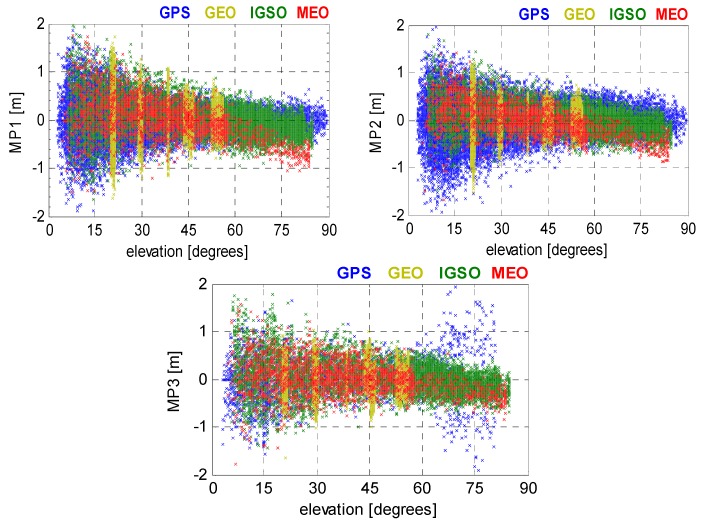
BeiDou and GPS code MP series against elevation angles observed from JFNG (DoY 081/2015).

Shown in Figure 1 are the MP series of BeiDou satellites observed on JFNG (Wuhan, China) with a Trimble Net R9 receiver. BeiDou satellites are grouped by three different orbit types, *i.e.*, GEO, IGSO, and MEO. For comparison reason, the MP series of GPS satellites tracked by the same receiver are plotted as well in Figure 1. As expected, signals observed at low elevation angles are more likely to be affected by multipath error. On the contrary, a high elevation satellite is less likely to cause multipath error. However, unexpected code biases (drifts), which are absent for GPS, are found in BeiDou MEO and IGSO satellites. The code bias variation reaches approximately 1 m at high elevation angles, particularly for the MEOs on the B1 signal. Similar variation and its characteristics have been well discussed in Wanninger and Beer [22]. Since the code bias variation was consistently observed on other stations equipped with different receivers and antennas, such bias thus was attributed to the transmitting satellites. In addition, anomalies can be found in the MP3 series of GPS (*i.e.*, code multipath effect on L5 signal), which show significantly larger values at high elevation angles. Similar code-phase divergences, which were attributed to satellite-internal signal reflections, had been earlier reported for GPS SVN49 and SVN62 in Spring and Dislssner [26]; Montenbruck *et al.* [27]; and Hauschild *et al.* 2012 [11].

To investigate the degree of linear dependence between MP and elevation, the Pearson correlation coefficient [28] was applied and computed as:

(2) $$\rho_{MP,E}=\frac{\sum MP\cdot E-\frac{\sum MP\sum E}{N}}{\sqrt{\left({\sum MP}^{2}-\frac{{\left(\sum MP\right)}^{2}}{N})({\sum E}^{2}-\frac{{\left(\sum E\right)}^{2}}{N}\right)}}$$

where E and N represent satellite elevation angle and the number of samples, respectively. The correlation coefficient $\rho_{MP,E}$ ranges from −1 to 1. A value of 1 or −1 implies that a linear equation describes the positive or negative relationship between MP and E perfectly, with all data points lying on a line. A value of 0 implies that there is no linear correlation between the two variables.

Table 1 lists the corresponding correlation coefficients for both GPS and BeiDou satellites. In general, the correlation coefficient under 0.3 represents low correlation, while the coefficient between 0.3 and 0.5 indicates normal. If the correlation coefficient exceeding 0.5 but less than 0.7, a medium correlation exists between two components. The correlation coefficient between 0.7 and 0.9 represents a high correlation. Additionally, if the correlation coefficients were beyond 0.9, there would be a strong correlation between two components. The coefficients in Table 1 demonstrate a medium correlation between MP and elevation for BeiDou IGSOs and MEOs, whereas the correlations of GPS and BeiDou GEO satellites are rather weak or even uncorrelated. Nevertheless, one should keep in mind that the GEOs are visible at almost constant elevation angles within restricted region (*i.e.*, Asia-Pacific region). It is unable to infer whether such code biases affect BeiDou GEO code measurements. Hence, the GEOs are excluded in the following context.

## 3. Observation Datasets

**Figure 2:**
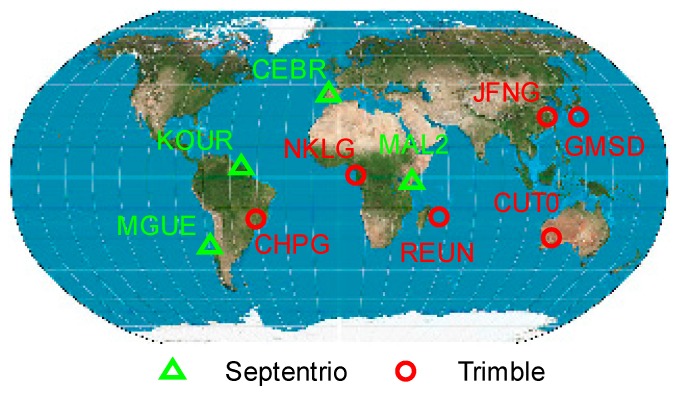
Distribution of the selected MGEX stations used in this study.

As of August 2015, the multi-GNSS experiment (MGEX) [29] offers a global network of approximately 70 stations of GNSS receivers capable to receive BeiDou signals. Most of these stations are equipped with Trimble NetR9 receivers providing triple-frequency BeiDou observations. However, only a few stations are equipped with other receiver types, such as Septentrio POLARX4/POLARX4TR/ASTERX3 and Leica GR25 receivers, providing dual-frequency observations on frequencies B1 and B2. To achieve a global coverage of receiving stations and to be able to compare different receiver types, six stations (CHPG at Cachoeira Paulista, Brazil, CUT0 at Perth, Australia, GMSD at Nakatane town, Japan, JFNG at Wuhan, China, NKLG at Libreville, Gabon, and REUN at Le Tampon, France) equipped with Trimble receivers and four stations (CEBR at Cebreros, Spain, KOUR at Kourou, French Guiana, MAL2 at Malindi, Kenya, and MGUE at Malargue, Argentina) equipped with Septentrio receivers were selected as shown in Figure 2. Data recorded on the first day of each month from January 2014 to October 2015 were used as the core datasets for this study. The datasets spanning almost two years facilitate analysis of a long-term variation of the BeiDou satellite induced code bias. However, it is worth mentioning that some of the data may be unavailable during this period due to station related reasons, e.g., new settled devices or upgrading issues, *etc*. Whatever the reason, an average of 13–14 days data is usable for each station.

**Figure 3:**
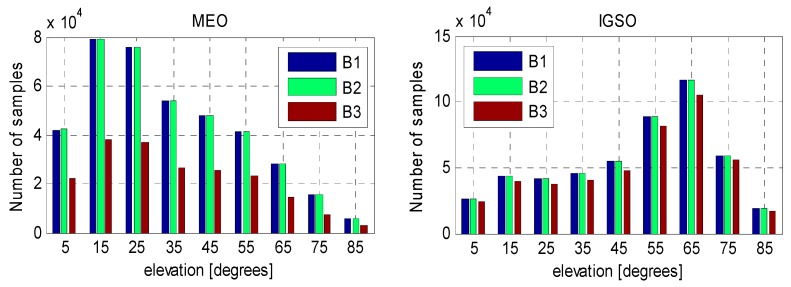
Number of MP samples against elevation bins with an interval of 10° for BeiDou MEO (**left**) and IGSO satellites (**right**).

Figure 3 shows the number of MP samples against elevation bins with an interval of 10° for BeiDou MEO and IGSO satellites. As already mentioned, stations equipped with Septentrio receivers provide only B1 and B2 signals and, thus, the number of MP3 for MEOs is much smaller than that of MP1 and MP2. As to IGSOs, the number of MP3 is comparable to that of MP1 and MP2 since the IGSO satellites are mostly tracked by triple-frequency Trimble receivers in Asia-pacific area. Furthermore, the number of MP samples is relative smaller at the lowest and highest elevation bins due to spatial configuration reason.

## 4. Improved Correction Model

As shown in Figure 1, the code bias varies linearly with elevation. To obtain the best fitting results, continuous piecewise linear functions were used to model the code-phase divergences. Note that the term continuous is used in the sense that the adjacent segments of the function share the same end point. Given a set of MP series, $\left(E_{i},MP_{i}\right)$, which can be divided into several consecutive segments, the following objective functions should be satisfied to find the best fits:

(3) $$\begin{array}{l} S=\sum_{j=1}^{m-1}\sum_{i=1}^{n_{j}}{\left(f_{j,i}-MP_{j,i}\right)}^{2}=\min\\ \begin{array}{cc} s\text{.t}. & f_{j}(a_{j})-f_{j+1}(a_{j})=0 \end{array} \end{array}$$

where

- i=1,2,⋯n: point index; n: number of points (samples);
- j=1,2,⋯m−1: segment index; m: number of nodes;
- $a_{j}$: elevation angle on a specific node (*i.e.*, the end point of *j*-th segment);
- $b_{j}$: MP value on a specific node;
- $f_{j,i}=f_{j}(E_{j,i},b_{1},b_{2},\cdots b_{m})$: fitted function used in the *j*-th segment.

Solving the normal linear system of Equation (3) with least squares method produces the MP values on all nodes. Then, the precision of estimates, *i.e.*, the root mean square (RMS) is derived as:

(4) $$RMS_{j}=\sqrt{\frac{\sum_{i=1}^{n_{j}}{\left(f_{j,i}-MP_{j,i}\right)}^{2}}{n_{j}-1}}$$

**Figure 4:**
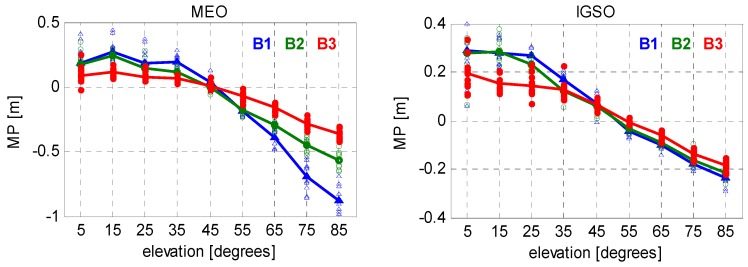
Piecewise linear MP models for BeiDou MEO (**left**) and IGSO satellites (**right**) on JFNG station using data from different days (during January 2014–October 2015).

**Figure 5:**
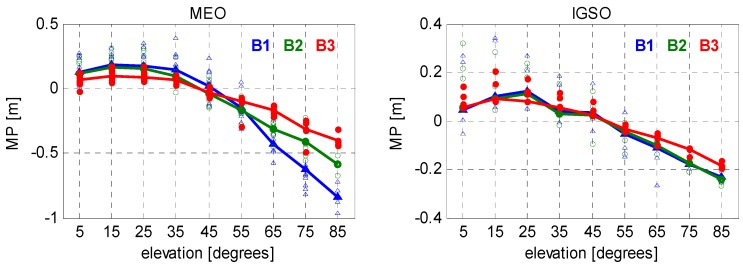
Piecewise linear MP models for BeiDou MEO (**left**) and IGSO satellites (**right**) using data from different stations (equipped with six Trimble receivers and four Septentrio receivers).

To ensure enough samples are used and to get reliable fitting functions, we selected the nodes to be separated by 10° of elevation, and the node values for the elevation angles range from 5° to 85°. Representative results of such continuous piecewise linear models are presented in Figure 4 and Figure 5. Figure 4 shows the results for JFNG, a MGEX station located in Wuhan, China, which is equipped with a Trimble NetR9 receiver. All available BeiDou data during the test period (*i.e.*, January 2014–October 2015) were used to model the code biases on B1, B2, and B3 frequencies. Scatterplots on each node represent the MP values of different days, which clearly reflects the variation of code bias with time. The solid lines connect the averaged MP values on consecutive segments. Likewise, the code biases estimated from all the selected stations on the same day (DoY 001/2015) were shown in Figure 5. Differently, scatter plots on the same node represent MP values of different stations, which gives us an intuitive impression of their consistency.

Obviously, such code biases are orbit type-, elevation-, and frequency-dependent, which is consistent with Wanninger and Beer [22]. The code measurements of MEO satellites tracked on B1 frequency at high elevations are seriously affected by the code bias, which reaches over 0.5 m when the elevation rises up to 70°. The code biases on B2 and B3, however, are much smaller than those on B1. As to the IGSO group of satellites, similar effects can be found among triple frequencies. However, the values of code bias as well as their frequency-dependent differences are much smaller compared to those of MEOs. In general, the code biases show a good consistency among different stations (equipped with different receivers) and different days and, thus, make it possible to be correctly modeled with limited data. Nevertheless, it is worth noting that slight differences do exist in the models if different samples are used.

**Figure 6:**
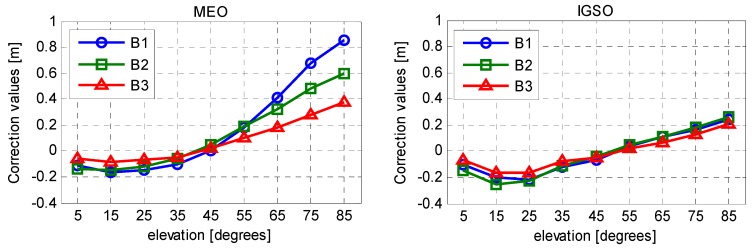
Suggested correction values of code bias (orbit type-, frequency-, and elevation-dependent).

**Figure 7:**
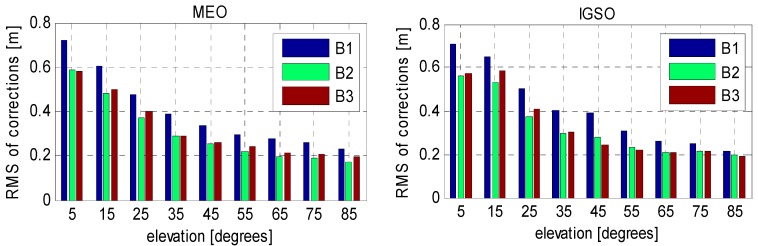
Suggested RMS of correction values (orbit type-, frequency-, and elevation-dependent).

Therefore, in order to obtain the best fitting model of code biases, MP series derived from all the selected stations during a period of almost two years were used to produce the correction model for BeiDou satellite induced code bias. In addition to the correction values, the corresponding precisions are also essential for the users to have a better understanding of their corrections. Notice that there is very large RMS-values at low elevation for the reason that signals observed at low elevation angles are more likely to be affected by multipath error from stations, which decrease the precision of estimation. Figure 6 and Figure 7 show the resulting correction values and their RMS for elevations range from 5° to 85° with a node separation of 10°. The final model parameters are summarized in Table 2.

To make use of them, correction values for elevations between 5° and 85° can be obtained by linear interpolation from the nearest two nodes (Equation (5)), whereas the corrections on nodes of 5° and 85° are simply used for the elevation bins of (0,5) and (85,90). Assuming the MP correction values at node points are uncorrelated and neglecting the interpolation error, the precision of a given point can be determined by the law of propagation of variance in Equation (5):

(5) $$\begin{array}{l} MP(e)=MP_{0}+(MP_{1}-MP_{0})\cdot \frac{e-E_{0}}{E_{1}-E_{0}}\\ \sigma_{MP(e)}^{2}={\left(\frac{E_{1}-e}{E_{1}-E_{0}}\right)}^{2}\cdot \sigma_{0}^{2}+{\left(\frac{e-E_{0}}{E_{1}-E_{0}}\right)}^{2}\cdot \sigma_{1}^{2} \end{array}$$

where $E_{j}$, $MP_{j}$ and $\sigma_{j}^{2}$(j=1,2) are, respectively, the elevation angles, MP values and the corresponding RMS on the nearest two nodes; MP(e) and $\sigma_{MP(e)}$ are the interpolated MP correction and RMS at the given elevation angle e.

**Figure 8:**
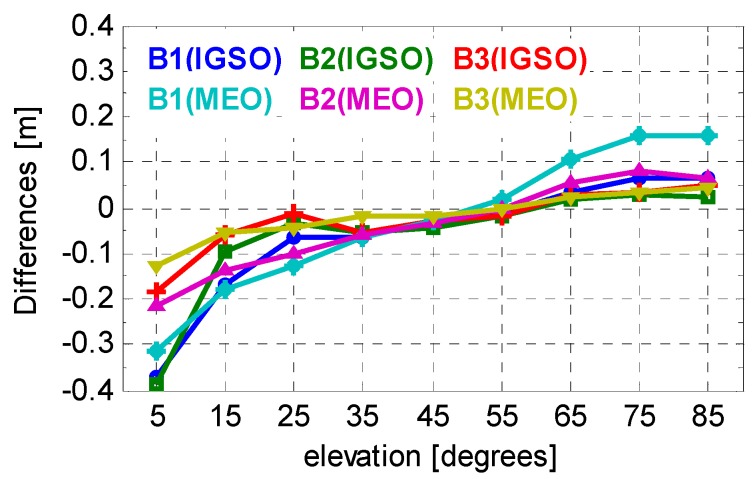
Differences of the code bias correction values compared with those of Wanninger and Beer.

Figure 8 shows the correction differences with respect to Wanninger and Beer [22]. In general, the differences are mostly within 0.1 m, which implies that our model agrees well with the existing one. However, the values of the differences at low elevation bins are much bigger, which reach 0.2–0.4 m. This is caused by the significantly larger noise level at low elevation angles. The precision of MP corrections as listed in Table 2 confirms this.

## 5. Validation of the Models

To verify the proposed correction model, absolute positioning techniques, such as standard point positioning (SPP) and PPP, are good choices since they both are affected by the satellite-induced code biases described above. It should be noted that, however, such code biases may be obscured by the residual ionospheric delay and broadcast ephemeris (*i.e.*, satellite orbits and clock corrections) errors, which are regarded as the dominating errors in SPP. Consequently, the code bias correction has marginal effect on the positioning results. To clearly demonstrate the effectiveness of the improved correction model, PPP tests were performed with three different processing schemes. In the first scheme, the satellite-induced code biases were kept without any treatment. In the second scheme, the traditional correction model provided in Wanninger and Beer [22] were used to remove the code biases. The improved correction model which contains correction values as well as precision indexes were applied for mitigating the same code biases in the last scheme. In the interest of brevity, “None”, “Traditional”, and “Improved” are used to denote the above-mentioned three different schemes throughout the rest of this article, if there is no additional explanation. The common processing strategies for PPP are summarized in Table 3. Precise BeiDou orbits and clock corrections provided by German Research Center for Geoscience (GFZ, Potsdam, Germany) were used. The positioning results were compared with the IGS weekly solutions [30]. In general, the reference coordinates have an accuracy of few millimeters.

### 5.1. Positioning Error

**Figure 9:**
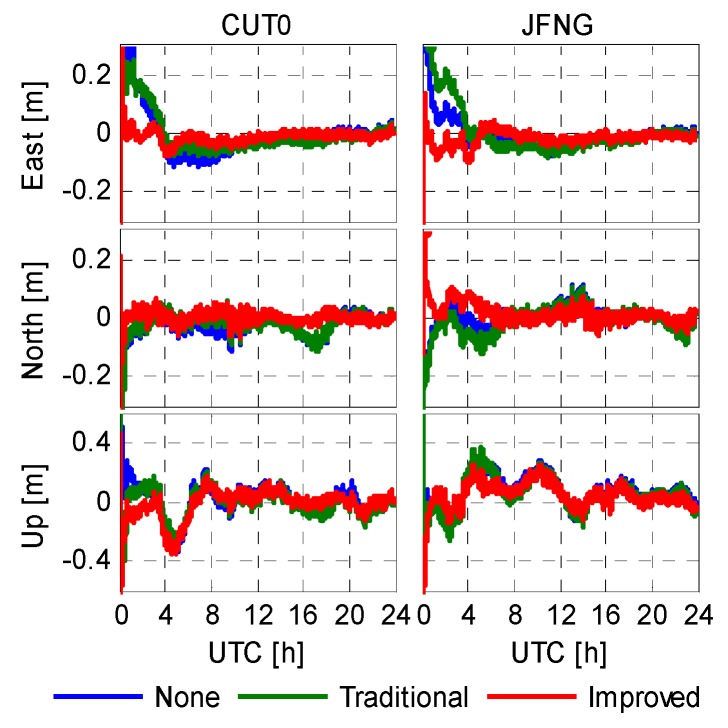
Epoch-wise PPP solutions of CUT0 and JFNG (DoY 047/2014).

**Figure 10:**
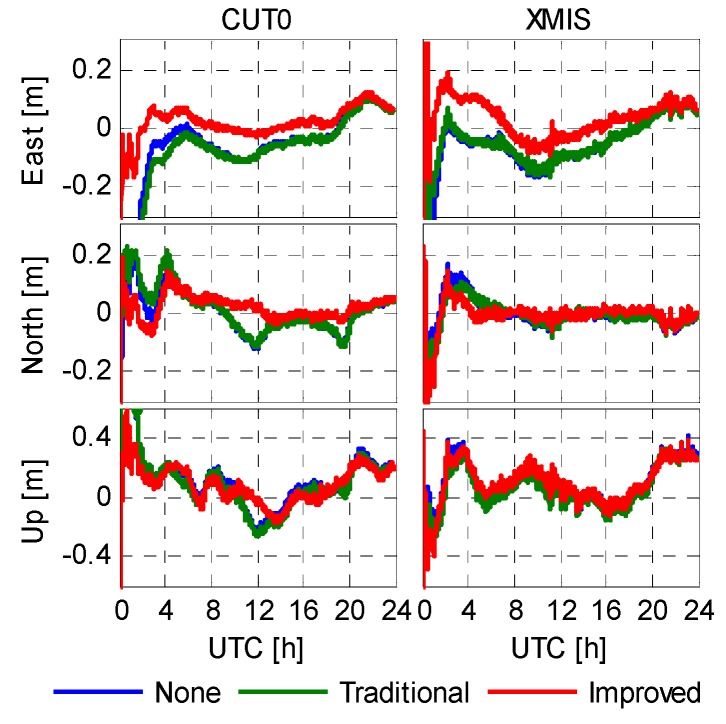
Epoch-wise PPP solutions of CUT0 and XMIS (DoY 130/2015).

The validation of the correction is verified by PPP solution using five stations: CUT0, JFNG, XMIS (Christmas Island, Australia), GMSD (Nakatane town, Japan), NNOR (New Norcia, Australia), during days 45–50 in 2014 and days 130–135 in 2015 while the XMIS and SIN1 (Singapore) stations were excluded from the code bias modeling. Two representative PPP solutions on different stations and different days are shown in Figure 9 and Figure 10. Figure 9 shows the epoch-wise positioning error of CUT0 and JFNG recorded on DoY 047/2014, and Figure 10 shows the positioning error of CUT0 and XMIS recorded on DoY 130/2015. Owing to the highly-weighted carrier phase, the positioning results are not seriously affected by the un-modeled code biases. In general, an accuracy of 0.1 in horizontal and 0.2 m vertically can be achieved after convergence with the current BeiDou constellation even if we leave the code biases uncorrected. By comparing the first two schemes, the code bias corrections with the traditional model does not really yield noticeable improvements in terms of positioning. Sometimes inaccurate correction values may even degrade the positioning accuracy. However, the third scheme outperforms the former two in terms of positioning accuracy as well as convergence speed. This implies that the improved model, which provides not only correction values but also stochastic information, greatly enhances PPP performance, particularly at the initial stage.

**Figure 11:**
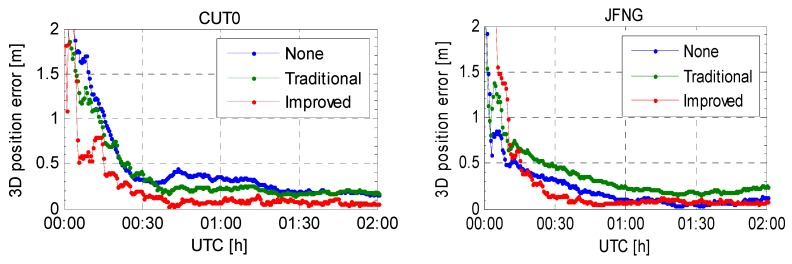
3D positioning error of the first two hours on CUT0 and JFNG (DoY 047/2014).

To gain more insight into the PPP convergence, the three dimensional (3D) positioning errors of the first few hours on CUT0 and JFNG are shown in Figure 11. At the initial stage, large differences can be observed for both CUT0 and JFNG, indicating that the positioning results are quite sensitive to the different processing schemes. After a short time convergence (15–30 min), the positioning accuracy of CUT0 is noticeably improved by the code bias corrections with the traditional model, whereas the situation is totally different for JFNG. Note that, the convergence time means obtaining a 3D positioning error less than one decimeter, which has been adopted by Li and Zhang [17]. The convergence time for five stations under three schemes and the average convergence time are shown in Table 4.

In fact, the correction values may be not accurate enough. The inaccurate corrections may even degrade the precision of code observations. However, as already mentioned, the stochastic information of the code bias corrections are completely missing in the traditional model. In other words, the precision of the code observables has been overestimated in this case. This will, unfortunately, lead to an uncertainty of their corrections, and eventually the positioning accuracy cannot be always enhanced. Compared against the former two schemes, solutions with the improved model show the best positioning performance with an accuracy of ~0.1 m, suggesting that a proper stochastic model for the corrections is critical. Reviewing the RMS of the code bias corrections in Table 2, we can find that the precision of the provided corrections range 0.2–0.7 m. Such an uncertainty is comparable to the assumed noise level of code measurements. Therefore, the actual precision of the corrected code observations can be reflected in an objective manner once the stochastic model of the corrections is taken into account.

### 5.2. Wide-Lane Ambiguity

**Figure 12:**
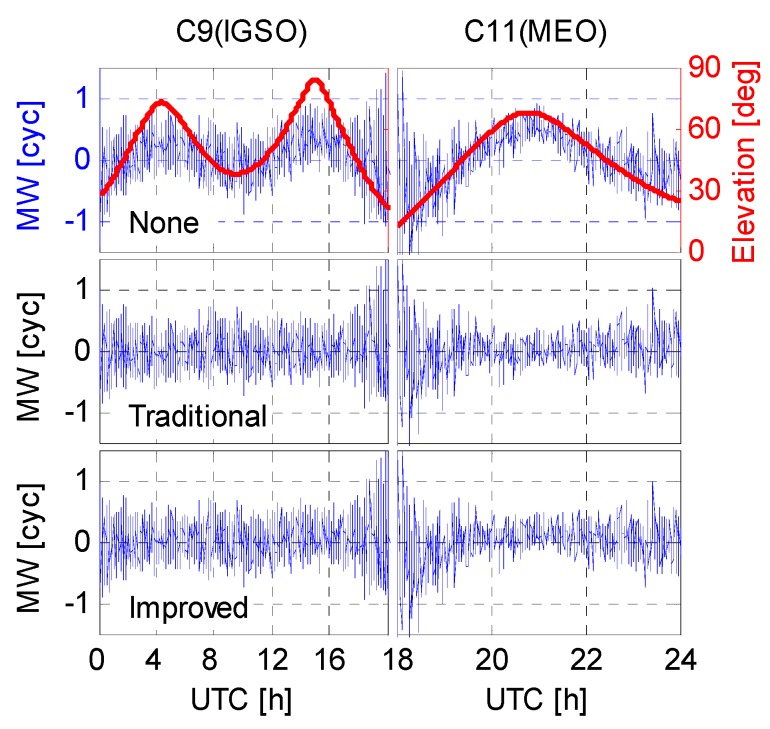
Time series of the derived MW value on XMIS (DoY 130/2015).

**Figure 13:**
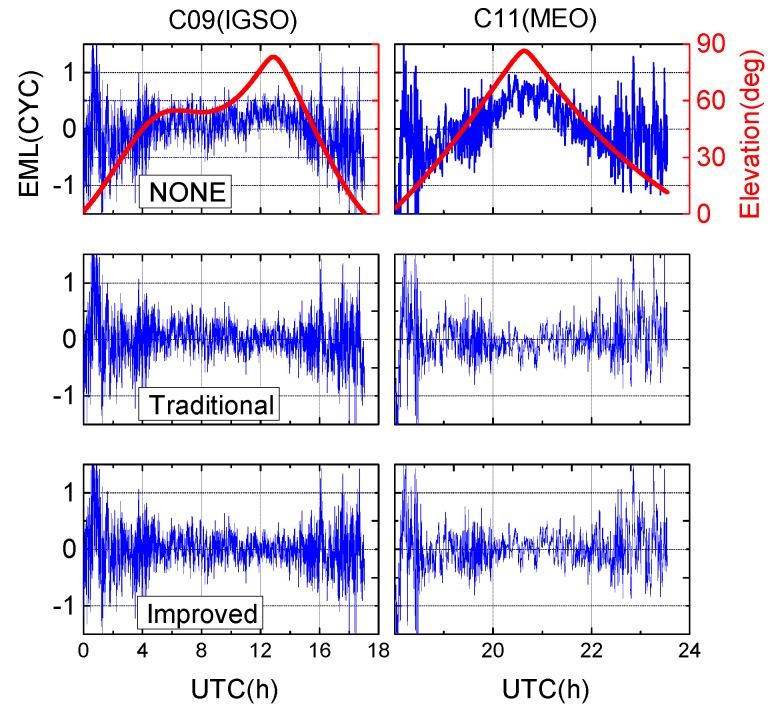
Time series of the derived EWL value on CUT0 (DoY 122/2015).

**Figure 14:**
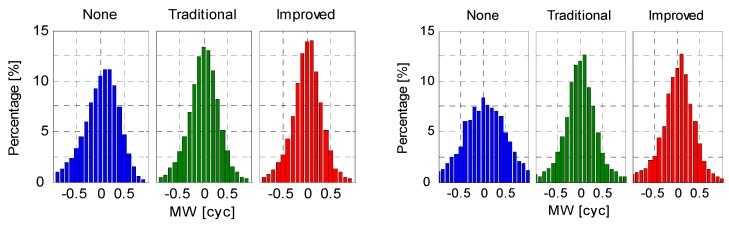
Histograms of all the derived MW values (**left**: IGSOs; **right**: MEOs) on XMIS (DoY 130/2015).

In addition to the positioning error, the wide-lane ambiguities, *i.e.*, the so-called Melbourne-Wübbena (MW) linear combination [36,37], were derived from the B1/B2 dual-frequency code and carrier phase measurements. Figure 12 and Figure 13 shows the WL ambiguity and EWL ambiguity of representative IGSO and MEO satellites with different code bias correction schemes. As shown in the upper two plots of Figure 12 and Figure 13, the WL and EWL values derived from original code observables reveal strong elevation-dependent systematic variations with amplitudes of half a wide-lane cycle, which prevents precise ambiguity determination and successful ambiguity resolution. After application of the code bias correction models, the systematic variations have been greatly removed, and the resulting MW series run much more stable and closer to the nearest integers. The fixing rate of MW ambiguities increases from 80.4% to 91.8%. Additionally, the MW values of all involved IGSO (C6, C7, C8, C9, and C10) and MEO (C11, C12, and C14) satellites were calculated and the statistics were given in Figure 14. Likewise, the MW derivations from the latter two schemes agree well with each other. This is reasonable since they have consistent code bias correction values as shown in Figure 8. Moreover, the distributions of the corrected MW values are much more concentrated compared to the one calculated from uncorrected code measurements. Obviously, the corrected MW values are more likely to be fixed, which definitely contribute to the subsequent undifferenced ambiguity resolution.

### 5.3. Code Residual

**Figure 15:**
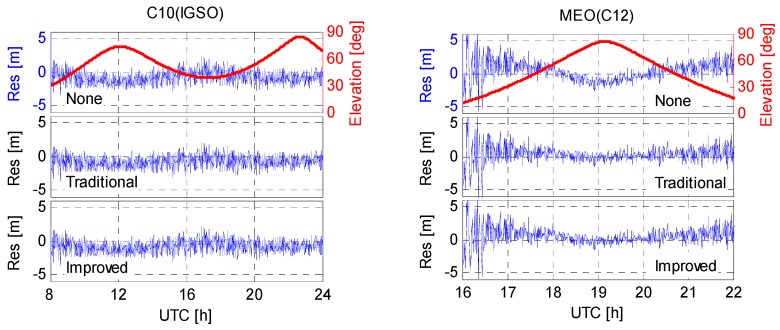
Time series of representative code residuals on XMIS (DoY 130/2015).

**Figure 16:**
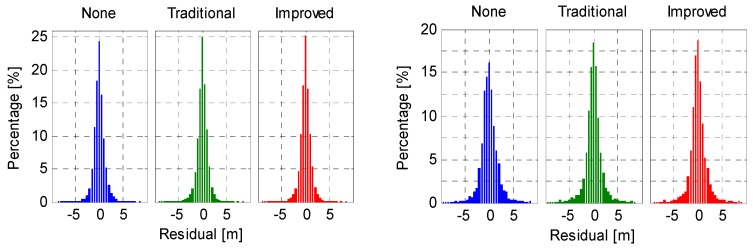
Histograms of all the code residuals (**left**: IGSOs; **right**: MEOs) on XMIS (DoY 130/2015).

Since the un-modeled code biases will be partly reflected in the residuals, the code residuals (*i.e.*, residuals of the B1/B2 ionosphere-free code combination) were analyzed as well. Taking the same station, for example, Figure 15 shows the code residuals (Res) of representative IGSO and MEO satellites on XMIS. As expected, the uncorrected elevation-dependent code bias variations are visible in the code residuals for both IGSO and MEO satellites. Such variations are stronger for MEOs since they are more seriously affected by the code-phase divergence. After application of the code bias correction models, the residuals run much more like zero mean random noises, which implies that most of the code biases have been well settled. Moreover, residuals of all the involved IGSO and MEO satellites were grouped together and the statistics were shown in Figure 16 and Table 5. The code residuals show a mean of 9 mm for the IGSO group satellites, and a zero mean for the MEO group satellites for all the three schemes. For IGSOs, residual distributions of the latter two schemes (*i.e.*, code biases corrected by the traditional and improved correction models) are slightly better than that of the first scheme (*i.e.*, none code bias corrections applied). As for the MEOs, code residuals show much better distributions in terms of standard deviation after mitigating the code biases with either the traditional or the improved correction models.

## 6. Conclusions

Different from the other GNSS systems, BeiDou (IGSO and MEO) code measurements are polluted by the so-called satellite-induced code biases, or code-phase divergences, or code bias variations. The multipath combination (MP), which is a geometry-free and ionosphere-free combination, was used to investigate the code bias variations. It was confirmed that such code bias variations are orbit type-, frequency-, and elevation-dependent. In general, the code measurements of MEO satellites tracked on the B1 frequency at high elevations are more likely to be affected by the code bias variations.

To mitigate these unexpected variations, an improved correction model was developed in this paper. Approximately two years data recorded on a globally distributed MGEX stations equipped with different receiver types were used. The continuous piecewise linear functions were employed to produce the correction values as well as their precision indexes. To obtain the best-fitting results, the elevation angles were limited to 5°–85° with a node separation of 10°. To make full use of the improved model, the stochastic information of the corrections were encouraged to take into account.

PPP tests were conducted with different code bias variation treatments to verify the effectiveness of the proposed model. Positioning results show that the traditional model does not really yield noticeable improvements. Sometimes it may even degrade the positioning accuracy due to its inaccurate stochastic model. However, the positioning accuracy, as well as convergence, were obviously enhanced by the improved model. This implies that the precision of the uncorrected code measurements and even the corrected code measurements with traditional correction model may be too optimistic. However, a more real and reliable stochastic model can be set up if the precision of corrections is taken into account. Moreover, the wide-lane ambiguities and code residuals were derived for comparison, which further confirmed the effectiveness of the proposed model.

## Figures and Tables

**Table 1:** Correlations between MP and elevation series.

	GPS	BeiDou		
		GEO	IGSO	MEO
**MP1**	−0.04	−0.01	−0.49	−0.61
**MP2**	0.08	0.00	−0.48	−0.60
**MP3**	0.12	−0.02	v0.39	−0.36

**Table 2:** Correction values and their precisions for BeiDou MEO/IGSO code measurements.

Elevation Nodes (°)	Correction Values (m)						RMS of Corrections (m)					
	MEOs			IGSOs			MEOs			IGSOs		
	B1	B2	B3	B1	B2	B3	B1	B2	B3	B1	B2	B3
5	−0.109	−0.140	−0.060	−0.101	−0.148	−0.065	0.721	0.588	0.580	0.709	0.564	0.576
15	−0.169	−0.148	−0.087	−0.203	−0.250	−0.162	0.605	0.480	0.499	0.651	0.532	0.582
25	−0.150	−0.121	−0.070	−0.222	−0.224	−0.168	0.476	0.373	0.401	0.500	0.371	0.409
35	−0.105	−0.062	−0.053	−0.123	−0.110	−0.078	0.388	0.291	0.290	0.403	0.297	0.303
45	0.004	0.047	0.022	−0.066	−0.043	−0.049	0.333	0.254	0.258	0.389	0.278	0.244
55	0.181	0.185	0.096	0.036	0.044	0.021	0.293	0.220	0.241	0.308	0.230	0.223
65	0.411	0.326	0.180	0.107	0.106	0.068	0.275	0.194	0.211	0.262	0.210	0.208
75	0.674	0.477	0.280	0.163	0.178	0.130	0.261	0.188	0.206	0.251	0.213	0.212
85	0.853	0.600	0.373	0.245	0.260	0.208	0.233	0.173	0.198	0.217	0.195	0.190

**Table 3:** PPP processing strategy.

Items	Models
Estimator (engine)	Kalman filter, TriP software [31]
Observations	B1/B2 code and carrier phase measurements
Sampling rate	30 s
Elevation cutoff	10°
Weighting scheme	Elevation dependent; 3 mm and 0.3 m for raw phase and code, respectively
Ionospheric delay	Eliminated by Ionosphere-free combination (s)
Tropospheric delay	Dry component: corrected with GPT model [32] wet component: estimated as random-walk process, GMF mapping function
Relativistic Effect	Applied
Station displacement	Corrected by IERS Convention 2010, including Solid Earth tide and ocean tide loading [33]
Satellite antenna phase center offset	Corrected with conventional PCO values from MGEX [34]
Receiver antenna phase center offset	Corrected by the same PCO values as GPS
Phase-windup effect	Corrected [35]
Receiver clock	Estimated, epoch-wise solution
Station coordinate	Estimated, epoch-wise solution
Phase ambiguities	Estimated, constant for each arc; float value

**Table 4:** Convergence time under three schemes for five stations.

Convergence Time (min)	Schemes		
	Uncorrected	Traditional	Improved
GMSD	125	108.5	29.5
CUT0	130	134	36
JFNG	76	136	32.5
XIMS	157	162	55
NNOR	130	127	33.5
Average	123.6	133.5	37.3

**Table 5:** Mean and standard deviation (STD) values of code residuals with different processing schemes (unit: m).

	IGSO			MEO		
	None	Traditional	Improved	None	Traditional	Improved
Mean	0.009	0.009	0.009	0.000	0.000	0.000
STD	1.166	1.155	1.152	1.802	1.730	1.740
